# Synthesis of Fluorescent Carbon Dots and Their Application in Ascorbic Acid Detection

**DOI:** 10.3390/molecules26051246

**Published:** 2021-02-25

**Authors:** Tengfei Wang, Hui Luo, Xu Jing, Jiali Yang, Meijun Huo, Yu Wang

**Affiliations:** 1 College of Food Science and Engineering, Shanxi Agricultural University, Taigu 030801, Shanxi, China; wangtengfei@sxau.edu.cn (T.W.); chsz519@163.com (H.L.); x.jing@163.com (X.J.); jiayang@sxau.edu.cn (J.Y.); 2 College of Veterinary Medicine, Shanxi Agricultural University, Taigu 030801, Shanxi, China

**Keywords:** fluorescent carbon dots, hydrothermal method, jujube, ascorbic acid

## Abstract

Water-soluble fluorescent carbon dots (CDs) were synthesized by a hydrothermal method using citric acid as the carbon source and ethylenediamine as the nitrogen source. The repeated and scale-up synthetic experiments were carried out to explore the feasibility of macroscopic preparation of CDs. The CDs/Fe^3+^ composite was prepared by the interaction of the CDs solution and Fe^3+^ solution. The optical properties, pH dependence and stability behavior of CDs or the CDs/Fe^3+^ composite were studied by ultraviolet spectroscopy and fluorescence spectroscopy. Following the principles of fluorescence quenching after the addition of Fe^3+^ and then the fluorescence recovery after the addition of asorbic acid, the fluorescence intensity of the carbon dots was measured at λex = 360 nm, λem = 460 nm. The content of ascorbic acid was calculated by quantitative analysis of the changing fluorescence intensity. The CDs/Fe^3+^ composite was applied to the determination of different active molecules, and it was found that the composite had specific recognition of ascorbic acid and showed an excellent linear relationship in 5.0–350.0 μmol·L^−1^. Moreover, the detection limit was 3.11 μmol·L^−1^. Satisfactory results were achieved when the method was applied to the ascorbic acid determination in jujube fruit. The fluorescent carbon dots composites prepared in this study may have broad application prospects in a rapid, sensitive and trace determination of ascorbic acid content during food processing.

## 1. Introduction

Fluorescent carbon dots (CDs) are a kind of monodisperse spherical-like carbon nano-material with a diameter of less than 10 nm and many functional groups on its surface [1]. In recent years, CDs have attracted much attention due to their excellent optical properties, good biocompatibility, low toxicity and responsive fluorescence quenching/enhancement properties. The research mainly focuses on developing a fast and straightforward synthesis method and the efficient use of CDs’ fluorescence characteristics, which have good prospects in food, materials, biology and other fields [2,3,4,5]. The synthesis of carbon dots can be generally divided into top-down and bottom-up methods [6]. The top-down approach includes laser irradiation, electrochemical synthesis and chemical oxidation [7,8,9]. The bottom-up process consists of a hydrothermal method, a microwave method and an ultrasonic method [10,11,12]. Researchers favor the hydrothermal method because of its simple operation, low cost and environmental friendliness [13].

Generally, the preparation of CDs by the hydrothermal method is to carbonize the precursor containing carbon by pyrolysis in a hydrothermal kettle. Various carbohydrates such as acids, sugars, proteins and other organic substances are used as carbon sources. Typically, there are a lot of natural biological materials that can be used as a carbon source to prepare biomass CDs, mainly in the following categories: (1) vegetable-derived biomass CDs, such as dehydrated mushrooms, lentils, coriander leaves, sweet potatoes and carrot juice; (2) fruit-derived biomass CDs, for example, pomegranate, papaya juice, bagasse, sugarcane juice, banana (Musa acuminate) juice, apple and citrus pectin hydrogel and (3) biomass CDs derived from other sources, for instance, grass, ginkgo leaves, bamboo leaves and other plants and black tea, soy milk and other beverages [14,15]. This type of carbon source is environmentally friendly, rich in sources, and low in cost, avoiding introducing other components in the synthesis process. However, one of the main problems with this method is low yield [16,17]. Doping of heteroatoms is a typical approach for modulating the electronic structure of CDs, and it is also a meaningful way to improve productivity. Among different dopants, the nitrogen source is sufficient. This is because its atomic size is similar to that of carbon, it is firmly electronegative, it has five valence electrons for chemical bonding and a lone pair of electrons are readily transferable to the π-orbitals of the sp^2^ carbon structures [18,19]. Recently, nitrogen-containing CDs have been synthesized using carbonization source heating in nitrogen-containing solvents. These substances can be used to synthesize nitrogen-doped CDs, such as dimethylformamide (DMF), ethylenediamine, amino acids, urea, dicyandiamide, amino silane, N-based polymers and various other *N*-containing natural materials [19]. Ascorbic acid, also known as Vitamin C, is a water-soluble vitamin that the body cannot synthesize or store and must take from the outside [20]. It is abundant in fruits and vegetables. As one of the essential vitamins to maintain the normal physiological function of the body, ascorbic acid is widely involved in complex metabolic processes, such as oxidation and reduction of the body, and plays a role in enhancing the immune function of the body and promoting the synthesis of collagen and mucopolysaccharides [21,22,23]. At present, there are many methods of ascorbic acid detection, each with its characteristics. The standard titration method is easy to operate, but the endpoint of titration is difficult to judge accurately. During the operation of spectrophotometric determination, it is necessary to constantly clean and replace the cuvette, which is cumbersome and time-consuming. High-performance liquid chromatography has high sensitivity, but a high analysis cost, and the price of detection equipment and the daily maintenance cost is relatively high [24,25]. Therefore, it is vital to explore an ascorbic acid detection method with high sensitivity, high accuracy, good reproducibility, a good linear relationship and suitable batch operation.

In this paper, citric acid and ethylenediamine were used as the raw materials to synthesize water-soluble CDs by the hydrothermal method. The feasibility of CDs macro-synthesis was investigated. CDs/Fe^3+^ composites were prepared by mixing CDs with ferric chloride in a particular proportion. A microplate reader measured the optical properties of CDs or CDs/Fe^3+^ composites. Based on the “on-off-on” principle, a batch, rapid and sensitive method for determining ascorbic acid content was established. Simultaneously, the procedure was applied to the determination of different jujube fruits with satisfactory results, which can provide a basis for the application of CDs/Fe^3+^ composites in food processing.

## 2. Results and Discussion

### 2.1. The Feasibility Analysis of CDs Synthesis and Their Macro-Preparation

**Figure 1:**
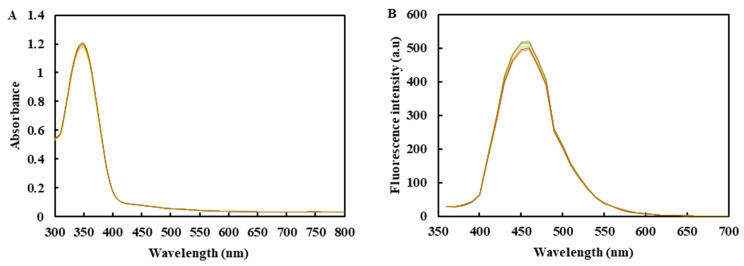
(**A**) Ultraviolet absorption spectrum of carbon dots (CDs) in each group. (**B**) The fluorescence emission spectrum of CDs in each group (Excitation wavelength at 360 nm).

Parallel experiments, storage stability experiments and extended synthesis were used to investigate the feasibility of the macro-synthesis of carbon dots. The maximum excitation wavelength of the five CDs groups was 360 nm and the RSD was 1.14% (Figure 1A). Under the excitation of 360 nm UV light, the CDs fluorescence emission spectrum (Figure 1B) showed that the fluorescence intensity of CDs in each group was similar, and the RSD was 2.06%. The peak position of the emission spectrum was almost constant in the error range. This result shows that CDs have excellent repeatability.

**Figure 2:**
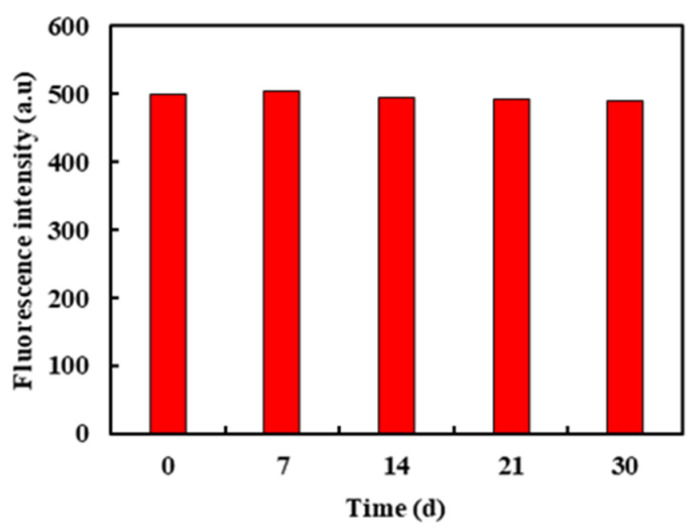
Storage stability of synthetic CDs.

As shown in Figure 2, with the extension of storage time, the CDs’ fluorescence intensity remains unchanged, which indicated that the CDs were relatively stable in the 30-day storage process.

**Figure 3:**
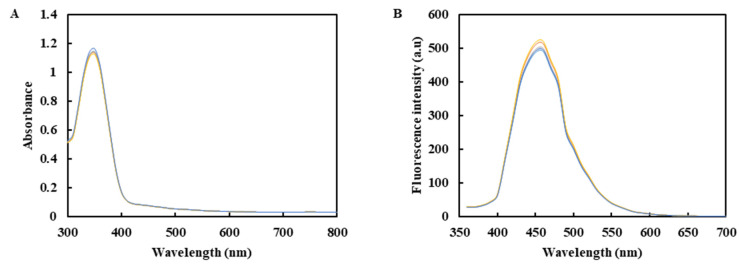
(**A**) Ultraviolet absorption spectrum of CDs. (**B**) The fluorescence emission spectrum of CDs (Excitation wavelength at 360 nm).

As shown in Figure 3, the maximum excitation wavelength of the expanded synthetic CDs material was 360 nm (Figure 3A), the optimal emission wavelength was maintained at about 460 nm (Figure 3B) and the RSD was 1.20% and 2.57%, respectively. The results show that the amplification synthesis has no significant effect on the CDs’ optical properties and has good reproducibility, which can effectively realize the macro-synthesis.

### 2.2. Characterization and Analysis of the Optical Properties of Carbon Dots

**Figure 4:**
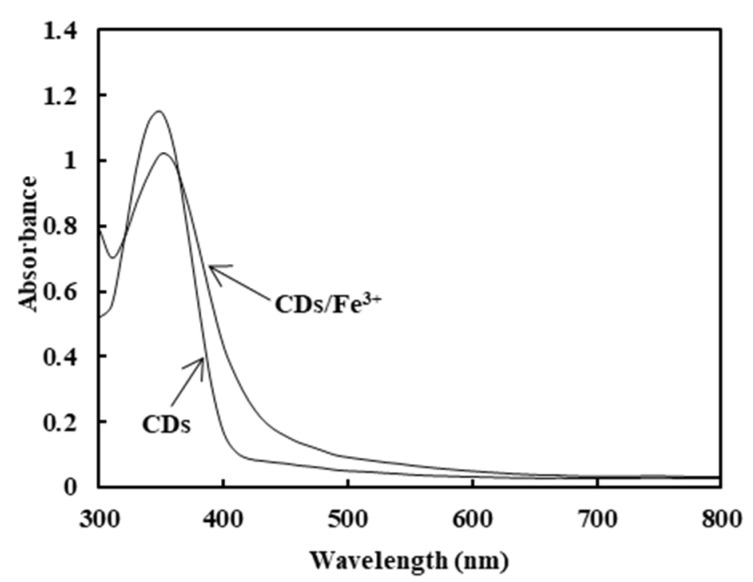
The UV absorption spectrogram of CDs and the CDs/Fe^3+^ composite.

The CDs and CDs/Fe^3+^ composite were characterized by UV–Vis absorption spectra, excitation spectra and emission spectra. The results are shown in Figure 4. The CDs characteristic absorption peak appears at about 350nm, which is mainly caused by the π-π^*^ transition [26]. The absorbance of the CDs/Fe^3+^ composite increased at 360 nm, which was caused by the addition of iron ions to increase the hybrid system’s conjugation degree.

**Figure 5:**
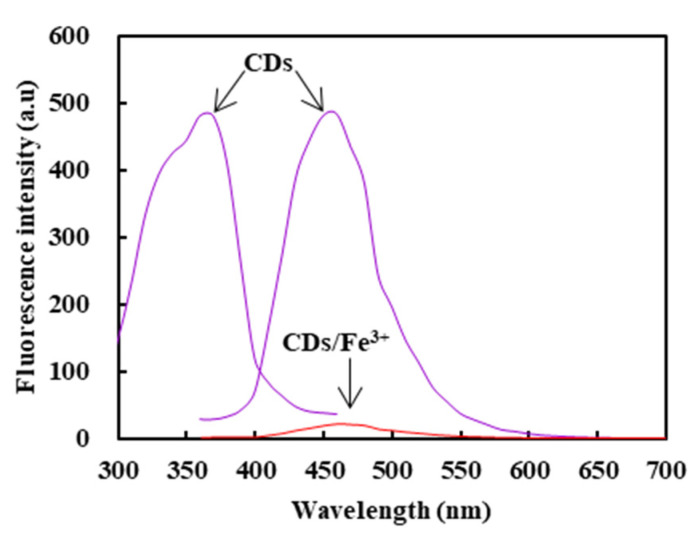
The fluorescence excitation spectrogram of CDs and fluorescence emission spectrogram of CDs and the CDs/Fe^3+^ composite.

The fluorescence excitation and emission spectra of CDs and the fluorescence emission spectra of the CDs/Fe^3+^ composite are shown in Figure 5. The maximum excitation wavelength and the emission wavelength of CDs were 360 nm and 460 nm, respectively. The fluorescence intensity of the CDs/Fe^3+^ composite was significantly reduced, which resulted from the quenching of CDs’ fluorescence by a ferric ion.

### 2.3. The Influence of pH on the Fluorescence of CDs

**Figure 6:**
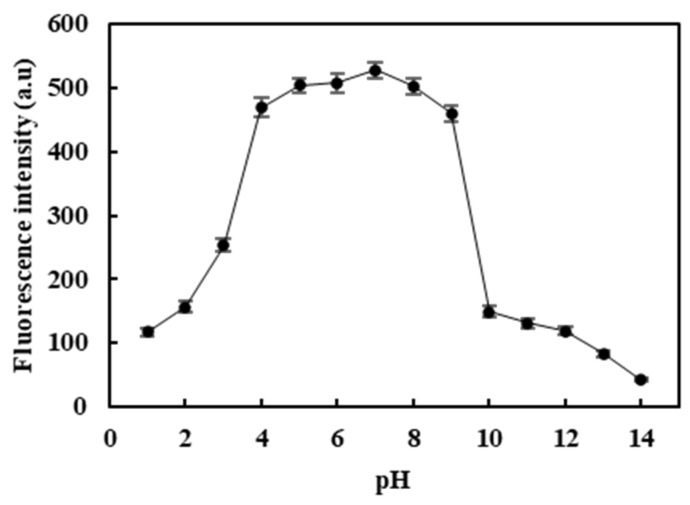
Effect of different pH values on the fluorescence intensity of CDs (excitation wavelength at 360 nm).

The carbon dots’ photostability was investigated by pH [27]. Figure 6 shows the pH effect (range from 1 to 14) on the CDs’ fluorescence intensity. It can be seen from Figure 6 that the fluorescence intensity of CDs was pH-dependent. The fluorescence intensity first increased with the increase of the pH value. When the pH value was between 4 and 10, the fluorescence reached the strongest intensity and was stable, and then the fluorescence intensity gradually decreased.The reason might be attributed to the triplet or surface structure changes of the CDs’ edge carbene structure [28]. In the condition of sharp acidity, the sawtooth points on the edge of the carbene structure of CDs were protonated by a hydrogen proton to form a reversible complex, which led to the destruction of the triplet carbene structure and fluorescence quenching [29]. The carbene structure of CDs was stable, and the fluorescence intensity was almost constant under neutral or weak base conditions. However, in a robust alkali solution, the molecular groups on the surface of the CDs were strongly affected, and the surface structure was destroyed, which led to fluorescence quenching [30].

### 2.4. Selectivity and Sensitivity of the CDs/Fe^3+^ Composite to Ascorbic Acid

**Figure 7:**
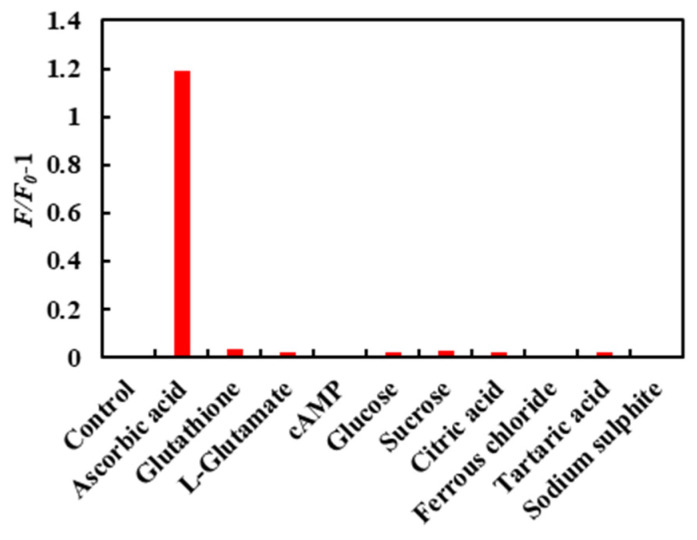
Effect of different biomolecules on the fluorescence intensity of the CDs/Fe^3+^ composite.

In addition to ascorbic acid, there are other components in the jujube. The influence of these components on the detection method was significant. Therefore, it was necessary to study other components in jujube fruit on the fluorescence quenching of carbon dots. Figure 7 shows the change of fluorescence intensity of the CDs/Fe^3+^ composite with different biomolecules. The fluorescence intensity of the CDs/Fe^3+^ composite solution was significantly enhanced when ascorbic acid was added (*F_0_* and *F* are the fluorescence intensity values of the CDs/Fe^3+^ composite before and after the addition of biomolecules). When adding other biomolecules, the fluorescence intensity changed little or not at all. The results show that the CDs/Fe^3+^ composite could specifically recognize ascorbic acid, which provides a new method for determining ascorbic acid.

**Figure 8:**
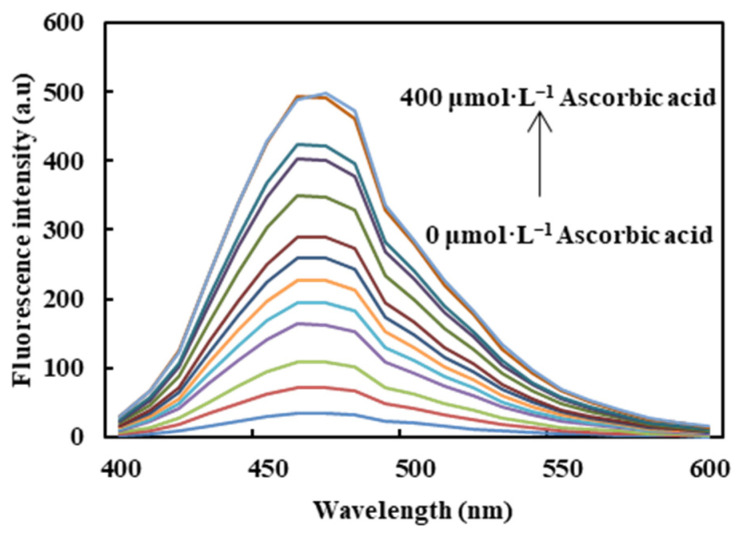
The effect of ascorbic acid concentration on the fluorescence intensity of the CDs/Fe^3+^ composite.

**Figure 9:**
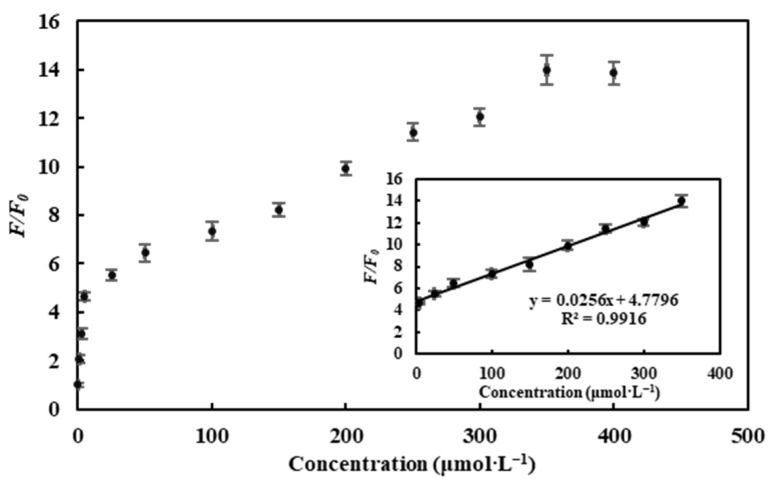
The plot of the fluorescence recovery factor (*F/F_0_*) versus concentrations of ascorbic acid (inset: a linear region).

As seen from Figure 8, the fluorescence intensity of CDs/Fe^3+^ gradually increased with increasing concentration of ascorbic acid. The plot of the fluorescence recovery factor *F/F_0_* (*F_0_* and *F* are the fluorescence intensity of the CDs/Fe^3+^ sensing system at 460 nm, in the absence and presence of ascorbic acid, respectively) against concentrations of ascorbic acid is shown in Figure 9. An excellent linear relationship between *F/F_0_* and the ascorbic acid concentration was obtained in the range of 5–350 μmol·L^−1^ with a correlation coefficient (R^2^) of 0.9916. The linear equation is expressed as *F/F_0_* = 0.0256[C] + 4.7796(μmol·L^−1^), where C is the concentration of ascorbic acid. The limit of detection (LOD) was estimated to be 3.11 μmol·L^−1^.

The limit of detection (LOD) was calculated based on the equation:

LOD = 3σ/k.

σ represents the standard deviation of blank signals of the CDs/Fe^3+^ sensing system (*n* = 10), and k denotes the slope of the calibration standard curve.

A comparison of materials, analytical range and detection limits between the present method and the other ways reported previously for ascorbic acid detection is listed in Table 1. Compared to previously reported methods for the quantitative determination of ascorbic acid, the present assay system we established offers a comparable or superior linear range and detection limit.

**Figure 10:**
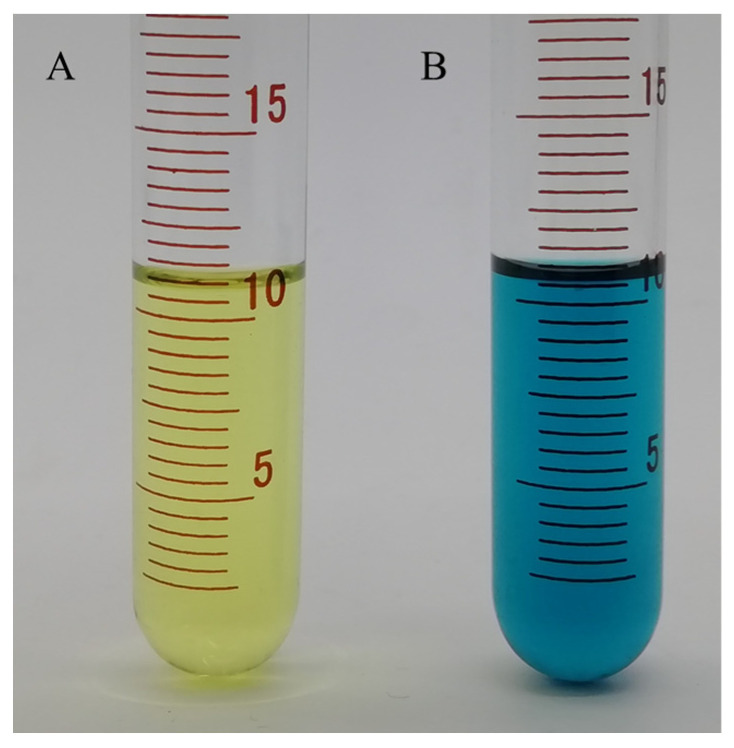
The color change after adding potassium ferricyanide to the CDs/Fe^3+^ solution with or without adding ascorbic acid (**A** tube contains a mixed solution without ascorbic acid and **B** tube contains a hybrid solution with ascorbic acid).

To further verify ascorbic acid’s effect on reducing ferric in CDs/Fe^3+^ composites, the method of Turnbull’s Blue was used to explore. Potassium ferricyanide solution (10 mmol·L^−1^) was added to the CDs/Fe^3+^ solution and the mixed solution of CDs/Fe^3+^ and ascorbic acid (350 mol·L^−1^), respectively. As shown in Figure 10, the hybrid solution of potassium ferricyanide and CDs/Fe^3+^ was yellow in the solution without ascorbic acid. In the solution containing ascorbic acid, the answer changed from yellow to blue. This phenomenon showed that the addition of ascorbic acid reduced the ferric in the CDs/Fe^3+^ composite solution to divalent iron ions and formed a complex with potassium ferricyanide [36]. The results show that the addition of ascorbic acid reduced the trivalent iron in the system to bivalent iron and restored the fluorescence of the CDs/Fe^3+^ composite solution.

### 2.5. Determination of Ascorbic Acid Content in Jujube Fruit

The results are shown in Table 2. The detected concentration by the CDs/Fe^3+^ composite method was consistent with those by the reference 2,6-dichloroindophenol method (*p* > 0.05), indicating that the CDs/Fe^3+^ composite method presented acceptable precision and accuracy and could be used for the rapid determination of ascorbic acid content in jujube fruit.

## 3. Materials and Methods

### 3.1. Reagents and Materials

CDs were synthesized in a polytetrafluoroethylene reaction kettle (Weihai Autoclave Co., Ltd., Wuhan, China). Ultraviolet–visible absorption spectra and fluorescence spectra were detected by a multifunctional microplate reader (SpectraMax^®^ i3x, Molecular Devices, Sunnyvale, Austria). Samples were prepared by centrifugation in a low-speed centrifuge (SC-3610, Anhui Zhongke Zhongjia Scientific Instrument Co., Ltd., Hefei, China). The sample was weighted by an analytical balance (AR224CN, Changzhou Auhaus Instrument Co., Ltd., Changzhou, China). The liquid was drawn through a micro-pipettor (Eppendorf, Hamburg, Germany).

The citric acid (98%), Ethylenediamine (98%), Ascorbic acid (99%), cAMP (99%) and Potassium ferricyanide (99%) were purchased from Aladdin biochemical technology Co., Ltd. (Shanghai, China). 2,6-dichlorophenolindophenol, Ferric chloride, Ferrous chloride, Glucose, Sucrose, l-glutamate, Glutathione, Sodium sulfite and Tartaric acid (Analytical grade) were obtained from Tianjin kaitong chemical reagent Co., Ltd. (Tianjin, China). Mu Zao, Li Zao and Huping Zao Dates were from the jujube garden in Taigu County, Shanxi Province, China. The water used in experiments was ultra-pure water.

### 3.2. CDs Synthesis and the Feasibility of Their Macro-Preparation

The CDs were synthesized by a hydrothermal method [37]. Briefly, the citric acid (as carbon source, 0.500 g) and ethylenediamine (as nitrogen source, 0.239 mL) were measured by an analytical balance and a pipette, respectively. The solution included an added 5 mL of ultra-pure water and was placed in a reactor at 180 °C for 4 h, then naturally cooled to room temperature. The original solution was filtered by a 0.45 μm microporous membrane and then freeze-dried to make a CDs powder and stored at 4 °C in the dark.

The feasibility of the macro-preparation of fluorescent carbon dots (CDs) was investigated by parallel experiments, storage stability experiments and scale-up preparation experiments [38,39,40]. Parallel experiments prepared five samples of CDs. Each sample of CDs was a group, for a total of 5 groups. A microplate reader measured the UV–Vis and the fluorescence emission spectra of CD samples. CDs were stored at 4 °C away from light for different times (0, 7, 14, 21 and 30 days), and the storage stability was investigated by detecting the change of fluorescence intensity. The scale-up preparation experiment was carried out to examine the feasibility of the scale-up preparation of CDs, and 0.500 g of citric acid and 0.239 mL of ethylenediamine were used as the control group. On this basis, the scale-up was 2, 4, 8 and 20 times, respectively. The UV–Vis absorption spectra and the fluorescence emission spectra of five groups of CDs were determined.

### 3.3. Preparation of CDs/Fe^3+^ Composites

A total of 104 mg FeCl_3_ was added into the 1 L 0.100 g/L CDs solution and the solution of the CDs/Fe^3+^ composite was prepared by magnetic stirring for 30 min.

### 3.4. Characterization of UV–Vis Absorption Spectra and Fluorescence Spectra

The synthetic CDs solution and the CDs/Fe^3+^ solution were added to each well using a pipette in a transparent 96-well plate (200 μL each well). The UV–Vis absorption spectra were measured from 300 nm to 800 nm with a scanning speed of 10 nm/step. The CDs and the CDs/Fe^3+^ composite solution (200 μL each well) were added to each cell using a pipette in a black 96-well plate. The fluorescence intensity and fluorescence emission spectra of CDs were measured by using a microplate reader. The excitation wavelength and emission wavelength of CDs were obtained by observing the change of fluorescence intensity. When determining the excitation wavelength, the emission wavelength was fixed, and when choosing the emission wavelength, the excitation wavelength was selected. The slit width was 10 nm.

### 3.5. Effect of pH on the Luminescence Properties of CDs

A total of 0.5 mol·L^−1^ HCL and 1.5 mol·L^−1^ NaOH were used to adjust the solution of CDs to the desired pH (range from 1 to 14), then a 200 μL solution was pipetted into the black 96-well plates. The microplate reader scanned the fluorescence intensity at 360 nm.

### 3.6. Determination of Ascorbic acid in Different Jujube Fruits

The extraction of ascorbic acid was performed with some modifications in the previous methodologies [41]. A total of 150 mL of metaphosphoric acid solution (5%) was added to ten grams of jujube fruits, and the mixture was mixed in a shaker for 10 min. Using a metaphosphoric acid solution (5%), the volume was completed to 200 mL. Next, using a microporous membrane (0.45 μm), the final solution was filtered. Lastly, the content of ascorbic acid was determined by the methods of 2,6-dichloroindophenol and CDs/Fe^3+^ detection.

2,6-dichlorophenolindophenol titration method: the prepared jujube solution was titrated with the calibrated 2,6-dichloroindophenol.

Ascorbic acid content = [V × (V_1_−V_0_) × ρ]/(V_S_ × m) ×100 (the unit: mg/100 g)

Among them, the ascorbic acid content indicated the number of milligrams contained in a 100 g sample and the unit is mg/100g. V is the total volume of the sample extract, and the unit is mL. V_1_ is the volume of dye consumed by titration, and the unit is mL. V_0_ is the volume of dye consumed by blank titration, and the unit is mL. Vs. is the volume of the sample solution, and the unit is mL. ρ is that the 1 mL dye solution was equivalent to the quality of ascorbic acid, and the unit is mL. m is the mass of the sample, and the unit is g.

The method of CDs/Fe^3+^ composite detection was as follows: 1 mL of the jujube sample solution was drawn into an EP tube by a pipette, and then the same volume of CDs/Fe^3+^ composite solution was added, mixing evenly, 200 μL of the sample was removed in a 96-well plate by a pipette and the excitation wavelength was 360 nm. The ascorbic acid content in jujube fruit was calculated according to the standard curve and the measured fluorescence intensity.

## 4. Conclusions

The water-soluble CDs solution was synthesized by the hydrothermal method using citric acid and ethylenediamine as raw materials, the CDs fluorescence quenching effect after adding ferric ions and the ascorbic acid fluorescence recovery principle. CDs/Fe^3+^ composites were synthesized by the interaction of CDs solution and ferric chloride solution. The material was specific for ascorbic acid and the content of ascorbic acid was calculated by quantitative analysis of the fluorescence intensity. Under the optimal conditions, the linear range of ascorbic acid detection was 5.0–350.0 μmol·L^−1^, and the detection limit was 3.11 μmol·L^−1^. This method was successfully applied to determine the ascorbic acid content in jujube fruit, and the results were satisfactory.

The CDs not only have the advantages of low material cost, easy availability, simple operation and large-scale preparation, but they can also adopt the enzyme labeling method to achieve rapid batch detection with a small amount of the sample. The material has good specificity and high sensitivity for ascorbic acid. Therefore, it is expected to provide a new idea for the trace determination of ascorbic acid during food processing, and it has good development and application potential.

## Figures and Tables

**Table 1:** Comparison of previously reported fluorescent methods for ascorbic acid detection with the present method.

Material	Analytical Range (μmol·L^−1^)	Detection Limit (μmol·L^−1^)	Reference
PNCQDs/Cr^6+^	5.0–200	1.35	[31]
N,S-CDs/Fe^3+^	10–200	4.69	[32]
CDs/Cu^2+^	100–2800	60	[33]
CDs-SiO_2_/Fe^3+^	0–70	3.17	[34]
Mn-CDs	3–140	0.9	[35]
CDs/Fe^3+^	5–350	3.11	This work

**Table 2:** Determination results of ascorbic acid in different jujube fruits (*n* = 5).

Sample	2, 6-Dichloroindophenol Method	CDs/Fe^3+^ Composite Method		
	Actual Measurements (mg/100 g)	RSD (%)	Actual Measurements (mg/100 g)	RSD (%)
Mu Zao	454.84 ± 14.18	3.12	458.02 ± 8.17	1.78
Huping Zao	286.32 ± 15.02	5.25	280.51 ± 9.91	3.53
Li Zao	377.48 ± 16.37	4.34	380.62 ± 8.63	2.27

## Data Availability

All data generated or analyzed during this study are included in this published article.

## Sample Availability

Samples of the compounds are available from the authors.
